# Role of Reductive versus Oxidative Stress in Tumor Progression and Anticancer Drug Resistance

**DOI:** 10.3390/cells10040758

**Published:** 2021-03-30

**Authors:** Kyung-Soo Chun, Do-Hee Kim, Young-Joon Surh

**Affiliations:** 1 College of Pharmacy, Keimyung University, Daegu 42691, Korea; chunks@kmu.ac.kr; 2 Department of Chemistry, College of Convergence and Integrated Science, Kyonggi University, Suwon, Gyeonggi-do 16227, Korea; 3 Research Institute of Pharmaceutical Sciences, Seoul National University, Seoul 08826, Korea; 4 Department of Molecular Medicine and Biopharmaceutical Sciences, Graduate School of Convergence Science and Technology, Seoul National University, Seoul 08826, Korea; 5 Cancer Research Institute, Seoul National University, Seoul 03080, Korea

**Keywords:** reductive stress, ROS, antioxidant, Nrf2, homeostasis, redox balance, cancer progression

## Abstract

Redox homeostasis is not only essential for the maintenance of normal physiological functions, but also plays an important role in the growth, survival, and therapy resistance of cancer cells. Altered redox balance and consequent disruption of redox signaling are implicated in the proliferation and progression of cancer cells and their resistance to chemo- and radiotherapy. The nuclear factor erythroid 2 p45-related factor (Nrf2) is the principal stress-responsive transcription factor that plays a pivotal role in maintaining cellular redox homeostasis. Aberrant Nrf2 overactivation has been observed in many cancerous and transformed cells. Uncontrolled amplification of Nrf2-mediated antioxidant signaling results in reductive stress. Some metabolic pathways altered due to reductive stress have been identified as major contributors to tumorigenesis. This review highlights the multifaceted role of reductive stress in cancer development and progression.

## 1. Introduction

Cellular redox homeostasis is essential for proper transmission of the intracellular signaling involved in normal physiological processes. However, an one-sided shift towards either oxidative or reductive state may cause alterations in the intracellular redox milieu. Imbalance of redox status can occur as a consequence of exposure to infectious agents (e.g., bacteria, viruses, parasites, etc.), radiation, and toxins, as well as in certain disease conditions. Although an optimal level of reactive oxygen species (ROS) is crucial for intracellular signal transduction for proper cellular functioning, excessive ROS production causes oxidative stress which is implicated in aging and also in the pathogenesis of many human disorders, including cancer, obesity, neurodegenerative diseases, and diabetes [1,2,3].

**Figure 1:**
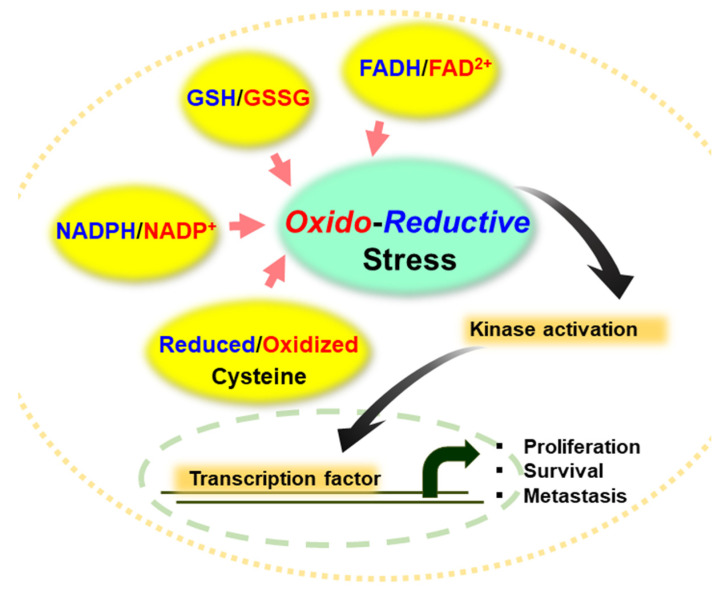
Representative antioxidant molecules and redox cofactors in regulating redox homeostasis. The ratio of GSH/GSSG, NADPH/NADP^+^ and FADH_2_/FAD^2+^ as well as the status of cysteine residues in proteins determines oxidative or reductive stress in normal and cancer cells, thereby modulating activity of kinases and transcription of genes involved in cell proliferation, survival, and metastasis.

Oxidative stress, defined as the shift of the balance between cellular oxidation and reduction potential towards the oxidizing part, is provoked when cellular antioxidant defense capacity is overwhelmed by massive production of ROS. Redox imbalance towards pro-oxidant conditions has long been considered to be implicated in the pathogenesis of tumor development and progression. In contrast, reductive stress is referred to as excessive production/accumulation of reducing equivalents, such as reduced glutathione (GSH), nicotinamide adenine dinucleotide phosphate (NADPH), nicotinamide adenine dinucleotide (NADH), and the free thiol group in the cysteine residues of proteins (Figure 1) [4]. An elevation in the ratio of GSH/GSSG, NADPH/NADP^+^ and NADH/NAD^+^, or overexpression of antioxidant enzymes can deplete ROS, which drives the cells to reductive stress [4]. Reductive stress is likely to be derived from intrinsic signals that enable cellular defense against pro-oxidative conditions. In addition, it is triggered by alterations in the production and/or utilization of reducing equivalents. The accumulation of superfluous reducing equivalents may disrupt normal cell growth responses, mitochondrial function, and cellular metabolism [5]. Recently, there has been increasing interest in the role of reductive stress in multistage carcinogenesis.

## 2. Reductive Stress as a Predisposing Factor for Tumorigenesis

Redox balance is not only essential for homeostasis in normal cells, but also important in the proliferation, progression, and survival of cancer cells [6]. Oxidative stress causes genomic instabilities and thereby triggers cancer metastasis and progression [6]. To counteract excess ROS, cancer cells constantly attempt to augment their antioxidant defense capacity [7,8]. Primary cells and tissues of mice overexpressing oncogenes as well as transformed epithelial cells at early stages of cancer progression exhibit lowered pro-oxidant status and higher antioxidant potential [9]. The resulting reductive environment stimulates proliferation and survival of transformed or cancerous cells and is therefore a promising therapeutic target for cancer prevention and treatment. In addition, cooperation among components that maintain the redox environment of cells can influence the therapeutic efficacy of anticancer drugs, especially those that exert cytotoxic effects through ROS generation [10].

**Figure 2:**
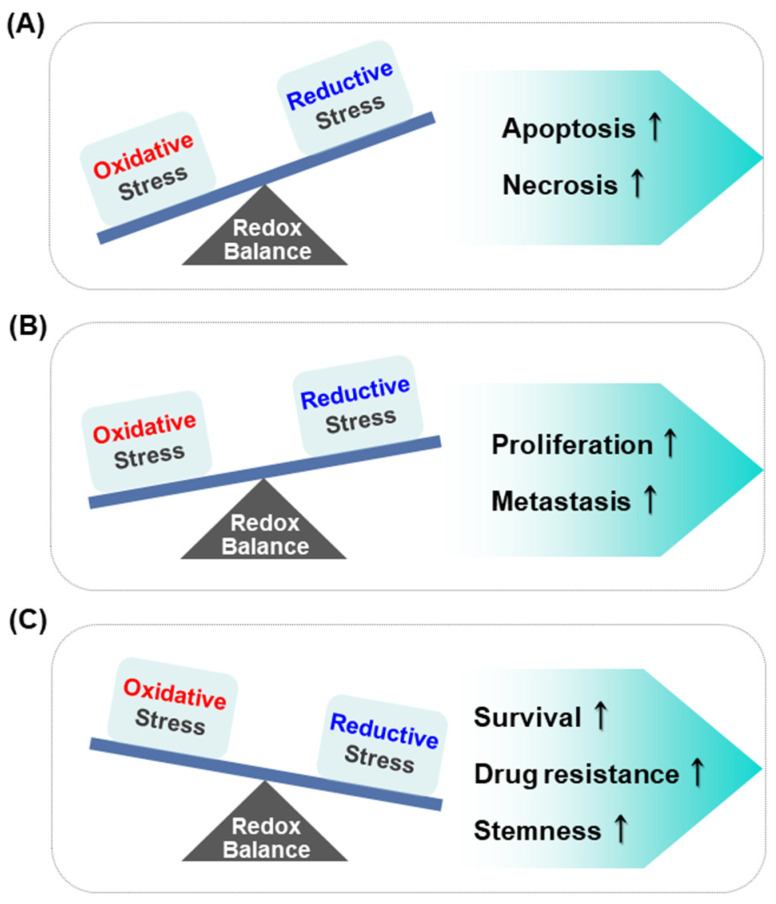
Effect of different levels of redox balance on the regulation of cellular processes in cancer cells. Oxidative stress, if extremely high, is cytotoxic (**A**), whereas moderate concentration of ROS induces the proliferation and metastasis of cancer cells (**B**). Reductive stress caused by low levels of ROS generation might lead to cancer cell survival, resistance to cancer therapy and stemness of cancer cells (**C**).

Although chemotherapeutic agents are intended to kill cancer cells through generation of ROS, this can eventually facilitate the development of drug resistance. It is noteworthy that a reductive redox environment fosters cancer migration and metastasis [11]. This suggests that tumors adapt to the high threshold of ROS by shifting their microenvironment to more reductive conditions. The effect of different levels of redox balance on the regulation of cellular processes in cancer cells is depicted in Figure 2.

## 3. Nrf2 as a Master Regulator of Cellular Redox Balance

Cells must maintain redox homeostasis by limiting the overproduction of ROS while properly operating antioxidant defense mechanisms to prevent or mitigate oxidative stress. Nuclear factor erythroid 2 p45-related factor (Nrf2) is a redox-sensitive transcription factor that functions as a master redox switch [12]. In unstressed cells, the Nrf2 protein is repressed by Kelch-like ECH-associated protein 1 (Keap1), which is a substrate adaptor for the Cullin-3 (Cul3)-dependent E3 ubiquitin ligase machinery. Keap1 has a distinct set of cysteine residues that are prone to oxidation or covalent modification by ROS or electrophiles, respectively. Modification of some of these sensor cysteines of Keap1 hampers its interaction with Nrf2 and Cul3 ubiquitin ligase complex [13]. As a result, Nrf2 dissociates from Keap1 and translocates into the nucleus, where it interacts with a small Maf protein. The resulting complex binds to the antioxidant response element (ARE) or electrophile-response element (EpRE), thereby transactivating the expression of a battery of genes encoding antioxidant enzymes and other cytoprotective proteins [14].

Cancer cells hijack signal transduction mediated by Nrf2 and make it hyperactivated through distinct mechanisms. The constitutively activated Nrf2 pathway enables cancer cells to survive with resistance to certain anticancer agents that exert tumoricidal effects mainly through ROS generation. It has been speculated that sustained accumulation or activation of Nrf2 confers upon a subset of premalignant or cancerous cells an advantageous environment for proliferation and metastasis by evading apoptosis and resisting therapeutic intervention [15]. Aberrant overactivation of Nrf2 has been observed in many different types of cancer, including lung cancer, breast cancer, ovarian epithelial carcinoma, endometrial cancer, and pancreatic cancer [9,16,17,18]. In addition, cancer patients with constitutively elevated levels of Nrf2 expression in tumor tissue show a lower survival rate [19]. Therefore, Nrf2 is considered as an important prognostic molecular marker for cancer, and Nrf2 overexpression/hyperactivation may reverse the phenotypic characteristics of cancer cells.

Autophagy is a lysosomal degradation pathway wherein cells adapt to stressful conditions by degrading proteins, aggregates, and cellular organelles. It is essential for the maintenance of cellular homeostasis, but recent studies have addressed its involvement in cancer [20]. It is known that autophagy is fine-tuned via the redox switch at the molecular level [21]. The stability of the Nrf2 signaling is regulated to some extent by the autophagy-related protein p62. p62 is a ubiquitin-binding protein that acts as a scaffold for several protein aggregates and triggers their degradation by the proteasomes or via the lysosomal pathway [22]. It is known that p62 directly interacts with Keap1, and thereby stabilizes Nrf2. Interestingly, expression of p62 is regulated by Nrf2, suggesting the existence of a positive feedback-loop in the p62-Keap1-Nrf2 axis [23].

**Figure 3:**
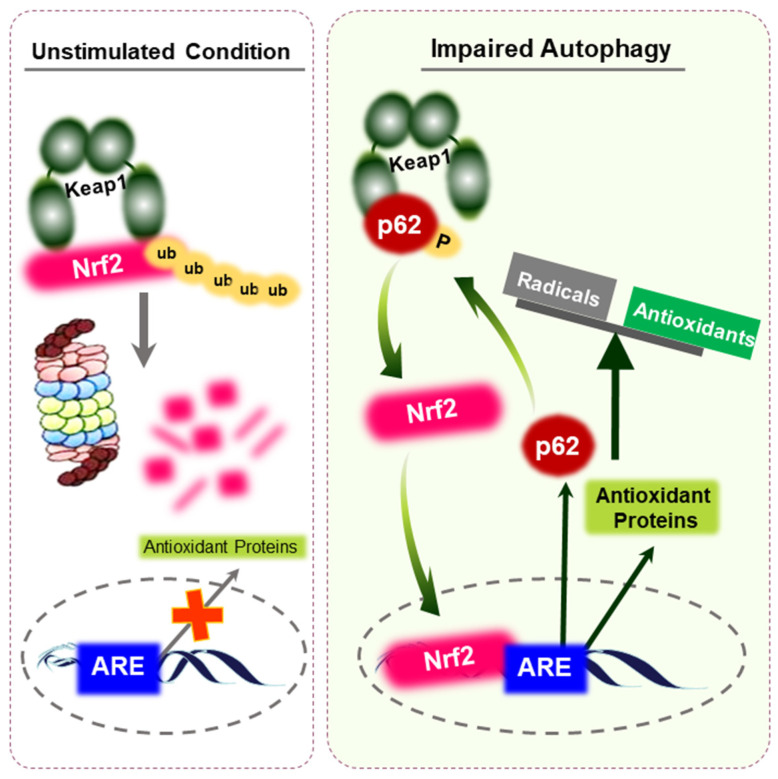
Activation of Nrf2 through p62 accumulation. Under unstimulated conditions, Nrf2 is constantly ubiquitinated by Keap1 and is rapidly degraded by the proteasomes. Upon impaired autophagy, defective accumulation of p62 results in the sequestration of Keap1. This leads to the release of Nrf2 protein and consequent potentiation of antioxidant capacity, provoking reductive stress. p62 is a target gene for Nrf2, implying that a positive feedback loop exists within the p62-Keap1-Nrf2 axis. Accumulated reductive stress might maintain the survival and resistance of cancer cells.

As p62 is a substrate for autophagy, the impairment of autophagy leads to its accumulation which, in turn, amplifies Nrf2 signaling. In muscle pathogenesis, accumulation of p62 results in sequestration of Keap1, with concomitant release of Nrf2 protein for nuclear translocation. The consequent activation of Nrf2 causes reductive stress, which is linked to cytoplasmic lamin aggregation [24]. The resulting lamin aggregates contribute to upregulation of p62 [24]. Aberrant p62 overexpression has been observed in certain types of cancer [25,26,27]. Accumulation of p62 promotes development of hepatocellular carcinoma through Nrf2 stabilization, leading to persistent transcription of target genes such as NQO1 and GST [28]. Direct interaction between p62 and Keap1 controls the Nrf2 signaling pathway to maintain redox balance. Therefore, augmentation of Nrf2 activity by autophagy is linked to reductive stress, which is schematically represented in Figure 3.

## 4. Role of Antioxidant Molecules in Reductive Stress Responsible for Cancer Progression and Drug Resistance

Although conventional chemotherapy has been successful to some extent, one of the major hurdles in cancer therapeutics is the development of multidrug resistance [29]. The resistance to chemotherapeutic drugs is acquired by various mechanisms. These include the alteration of drug targets, activation of drug efflux pumps, suppression of apoptosis-related factors, and enhancement of DNA repair capacity [30]. In addition, the simultaneous use of antioxidants and anticancer agents may attenuate the therapeutic efficacy by inducing the expression of enzymes that can inactivate or detoxify ROS-generating and alkylating cancer drugs [31]. Hence, the expression of antioxidant enzymes is likely to contribute to the maintenance of a redox environment which is favorable for the development of drug resistance. The next section will describe the role of some antioxidants and cellular antioxidant enzymes in regulating reductive stress for cancer development and drug resistance.

### 4.1. Glutathione (GSH)

GSH is a ubiquitous, nonprotein thiol antioxidant that plays a pivotal role in intracellular antioxidant defense and elimination of electrophilic toxicants [32]. The enzymes involved in the aforementioned processes include glutathione reductase, glutathione peroxidase, and glutathione *S*-transferase (GST). Although GSH has cytoprotective functions in normal cells, it contributes to resistance to anticancer drugs, many of which are alkylating or ROS-generating agents [33]. For instance, arsenic trioxide (As_2_O_3_) has been used for the treatment of leukemia and some solid tumors. Its therapeutic effects are attributed to ROS it generates. In androgen-independent prostate cancer (PC-3 and DU-145) cells, the combined treatment of As_2_O_3_ with a GSH-depleting agent, l-buthionine-sulfoximine (BSO) effectively inhibited cell growth [34]. The therapeutic efficacy of As_2_O_3_ combined with BSO was also verified in an orthotopic mouse model of prostate cancer [34]. In contrast, exposure of human breast cancer cells (MCF-7 and T47D) to exogenous GSH and another thiol antioxidant, *N*-acetyl-l-cysteine (NAC) dampened the antiproliferative activity of some anticancer drugs [35].

The redox cycling of doxorubicin is known to generate ROS, which accounts for its cytotoxicity towards tumor cells. Therefore, increased detoxification or inactivation of ROS or an active form of doxorubicin by GSH and GSH-dependent enzymes is considered to be implicated in resistance to this cancer drug. Cisplatin is another chemotherapeutic agent commonly used for the initial treatment of nonsmall cell lung cancer (NSCLC) [36]. It has been shown that GSH expression is increased in NSCLC, resulting in resistance to cisplatin treatment [36]. In addition, exogenous GSH could induce resistance to cisplatin in A549 cells derived from NSCLC by inhibiting apoptosis, inactivating mitochondria-mediated signaling pathways, and promoting expression of efflux proteins, such as multidrug resistance protein 1 (MRP1) [37]. Moreover, transient expression of P-glycoprotein (P-gp) was observed during the growth of multicellular tumorspheres of prostate cancer cells, which was associated with the escalation of GSH levels and reduced ROS generation [38]. Furthermore, intracellular ROS accumulation upon treatment with the GSH synthesis inhibitor BSO or ROS-generating agents has been shown to downregulate P-gp expression via the activation of receptor tyrosine kinase signaling [38].

Although the role for GSH in drug resistance has been well defined, there are some conflicting findings. Epidermal growth factor receptor (EGFR) inhibitors, such as erlotinib, are used in the treatment of EGFR-driven lung cancer. An acquired resistance to erlotinib is associated with EGFR T790M mutation. Thus, lung cancer patients harboring T790M mutation may not respond well to erlotinib [39]. Erlotinib-resistant cells have lower expression of GSH-synthesizing enzymes such as glutamate-cysteine ligase catalytic subunit, glutathione synthase, and glutathione reductase. Furthermore, GSH levels were significantly reduced in lung cancer cells expressing EGFR T790M mutation [39]. Decreased expression of GSH-synthesizing enzymes was found to be responsible for low GSH levels in resistant cells, which was attributed to the repression of Nrf2 transcriptional activity [39].

### 4.2. Manganese Superoxide Dismutase (MnSOD)

Superoxide dismutases (SODs) are metalloenzymes that catalyze the dismutation of superoxide anions to hydrogen peroxide. These enzymes are ubiquitously expressed in both eukaryotes and prokaryotes. SODs utilize metal ions such as copper (Cu^2+^), zinc (Zn^2+^), manganese (Mn^2+^), and iron (Fe^2+^) as cofactors. SOD enzymes have different subcellular localization and unique physiological functions. For instance, MnSOD activity is crucial for controlling the amount of ROS in the mitochondria. Fluid shear stress triggers apoptosis of circulating breast cancer cells by elevating the mitochondrial production of superoxide anions [40]. High levels of MnSOD in the mitochondria scavenge the superoxide anions induced by shear stress, which enables the circulating metastatic cells to resist apoptosis [40].

Notably, upregulation of MnSOD enhances the malignancy of tumor cells by sustaining the Warburg effect through activation of the AMPK pathway [41]. In aggressive breast cancer subtypes, the enhanced expression of MnSOD was strongly correlated with an increase in AMPK activation, as assessed by both the phosphorylation on the active site (Thr172) and phosphorylation of the downstream target, acetyl CoA carboxylase. Restricting MnSOD expression or inhibiting aberrant AMPK hyperactivation suppresses the metabolic switch in a way to reduce the viability of transformed cells [41]. Results from clinical studies have shown that the MnSOD/AMPK pathway is active in an advanced stage and aggressive breast cancer subtypes [41]. This appears to be achieved by creating a cellular environment that is conductive to reduced ROS in mitochondria of breast cancer stem cells (CSCs) [42]. Activation of the mammalian target of rapamycin (mTOR) is one of the most frequent events in human malignancies and is critical for sustaining the self-renewal ability of CSCs. Inhibition of mTOR activity by rapamycin suppressed the self-renewal of breast CSCs treated with ionizing radiation, which was associated with decreased MnSOD activity and increased ROS generation. [42]. Our recent studies have demonstrated that MnSOD is involved in migrative capability and clonogenicity of human breast cancer MCF-7 cells [43].

### 4.3. Glutathione S-Transferases (GSTs)

GSTs are detoxification enzymes that catalyze the conjugation of GSH to a variety of exogenous and endogenous electrophilic compounds. GSTs overexpressed in various malignancies play roles in the development of resistance to chemotherapeutic agents [44]. It has long been known that the expression of GST-α is elevated in many human tumors, relative to their matched normal tissues [45,46]. The chemotherapeutic agent, cisplatin, is used in the treatment of patients with head and neck cancer (HNC). Although untreated patients have relatively excellent response to cisplatin, those treated for relapsed or recurrent disease usually have poor treatment record with only a 20–30% response rate [47]. Cisplatin resistance in HNC is mediated by a number of different mechanisms, including drug transport/excretion, upregulation of DNA repair enzymes, and overexpression of antiapoptotic proteins such as Bcl-2 as well as induction of xenobiotic detoxification enzymes such as GST-π [48]. In particular, GST-π amplification is frequently observed in HNC cell lines and in tumors poorly responsive to cisplatin-based chemotherapy [49]. It has been reported that overexpression of genes such as *BRCA1*, *p53*, *p21*, *GST*, *MDR1*, and *TOP2A* is associated with the resistance of MCF-7 cells to doxorubicin [50]. Overexpression of these genes may hence predict the acquired resistance in breast cancer [50]. Tumorspheres derived from the A549 NSCLC cell line overexpressed various stem cell markers such as CD44, CD133, Sox2, and Oct4 [51]. In these cells, the expression of several drug resistance proteins, including lung resistance-related protein, GST-π, and MRP-1, was also significantly enhanced [51].

### 4.4. Heme Oxygenase (HO)

HO catalyzes the rate-limiting step in heme catabolism to generate carbon monoxide (CO), biliverdin, and free iron. At least two isoforms of HO have been identified in mammals: inducible HO-1 and constitutively expressed HO-2. HO-1 lacks cysteine residues, while HO-2 contains three Cys-Pro signatures, known as heme regulatory motifs (HRMs). These motifs are known to control the processes related to iron and oxidative metabolism in organisms [52]. The total expression of HO-2 does not change according to the intracellular redox state, but the cysteine residues in the HO-2 protein undergo a change in the thiol/disulfide state [52]. Oxidized HO-2 with Cys^265^ of HRM1 and Cys^282^ of HRM2 linked via intramolecular disulfide bond bind heme with high affinity [52]. However, under reducing conditions, the lower affinity dithiol form of HO-2 predominates, which gives rise to an increase in intracellular free heme [53]. Of note, some cancer cells have been shown to exhibit high levels of heme, a crucial cofactor for complexes of the mitochondrial electron transport chain, which allows them to sustain oxidative phosphorylation [54]. Therefore, reductive stress prevents the disulfide formation in the HRMs of HO-2, which may account for the elevated accumulation of heme in cancer cells.

Recently, Kim et al., reported that HO-2 could be a novel biomarker of tumor-initiating cells derived from human lung cancer and also a therapeutic marker for these cells [55]. Thus, genetic inhibition of HO-2 suppressed growth of tumor-initiating cells in the xenograft model [55]. Compared with HO-2, the inducible enzyme HO-1 appears to be less susceptible to redox modulation due to lack of a cysteine residue. However, aberrant overexpression of HO-1, in conjunction with hyperactivation of its principal regulator Nrf2, is involved in tumor growth, metastasis, and angiogenesis as well as resistance to anticancer therapy [56].

## 5. Role of Metabolic Pathways in Linking Reductive Stress to Tumorigenesis

Metabolic reprogramming is a hallmark of cancer, and recent studies highlight the metabolic flexibility of cancer cells [57]. The altered metabolic characteristics of cancer cells, such as dysregulated aerobic glucose metabolism, glutaminolysis, and fatty acid synthesis, affect cellular redox homeostasis. As mentioned earlier, changes in the redox status are associated with therapeutic resistance in cancer cells [57]. Higher ROS generation is balanced by an increase in antioxidant mechanisms that allow cancer cells to survive in a pro-oxidant environment [58]. Protection against oxidative stress is considered to be one aspect of metabolic reprogramming that supports cancer cell fitness [59,60]. Loss of extracellular matrix contact substantially alters their metabolic activity, leading to increased production of mitochondrial ROS.

Mitochondria represent a major source of both ROS and NADPH, and contain their own antioxidant systems. A growing body of evidence indicates that malignant progression of tumors is characterized by the occurrence of multiple alterations, wherein specific metabolic pathways are linked to the synthesis of essential building blocks for anabolic metabolism, such as amino acids, lipids, and nucleotides. Some of the energetic substrates involved in these pathways can also be redirected to specific metabolic routes to generate not only antioxidant molecules (e.g., GSH and NADPH) but also reducing cofactors (e.g., NADH and FADH) that can be readily used to maintain or restore adequate redox homeostasis [60,61,62].

### 5.1. Fatty Acid Oxidation (FAO)

**Figure 4:**
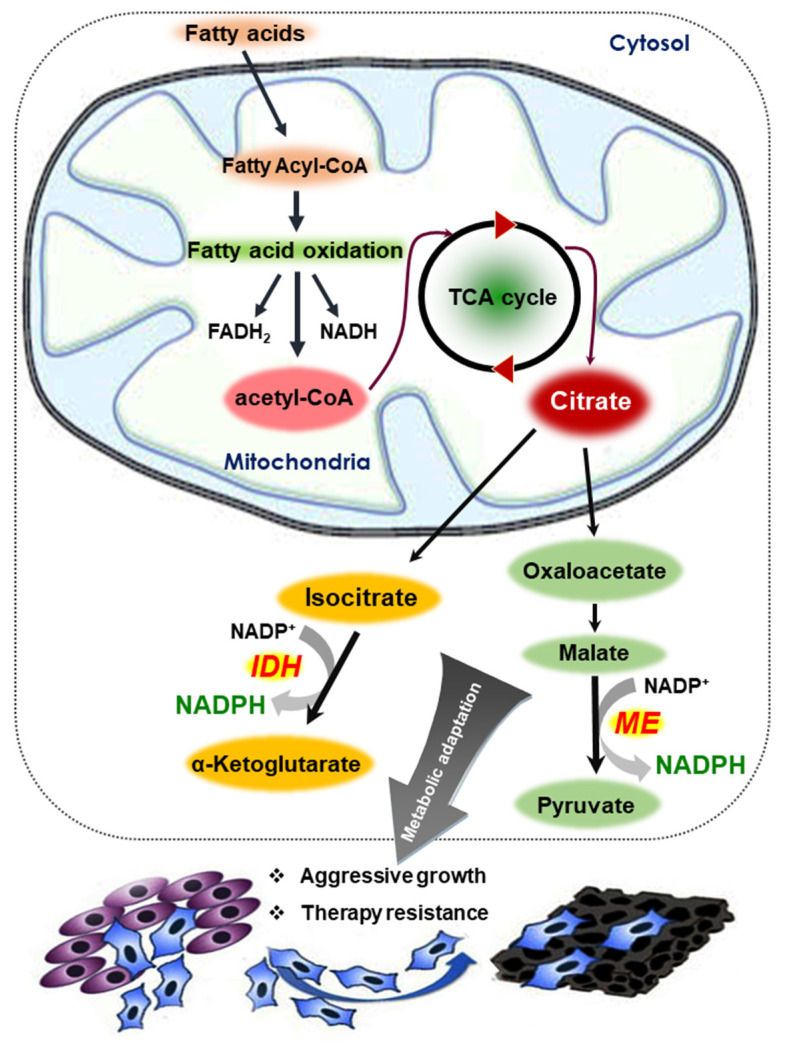
Mechanism of NADPH production from fatty acids. Acetyl-CoA generated as a consequence of FAO enters the TCA cycle and generates citrate. After export into the cytosol, citrate is funneled into metabolic reactions catalyzed by the malic enzyme (ME) and isocitrate dehydrogenase 1 (IDH1). During these reactions, large amounts of NADPH These enzymes ultimately produce are produced. Changes in redox balance within the tumor microenvironment allows cancer cells to become more aggressive and resistant to therapeutic agents.

FAO is the process by which fatty acid fuel molecules are broken down in the mitochondria to generate NADH/FADH_2_ and acetyl-CoA. Acetyl-CoA enters the tricarboxylic acid (TCA) cycle and generates citrate, which is exported into the cytosol and funneled into metabolic reactions catalyzed by the malic enzyme (ME) and isocitrate dehydrogenase 1 (IDH1). These enzymes ultimately produce large amounts of NADPH [63]. NADPH provides reducing power not only for antioxidant systems, but also for many biosynthetic reactions. It has been postulated that NADPH is the critical limiting factor for cancer cell proliferation (Figure 4). Detachment of mammary epithelial cells from extracellular matrix causes an ATP deficiency, which leads to an increase in cellular ROS and a decrease in GSH. Treatment with antioxidants was found to promote the survival of cells through stimulation of FAO [64]. In another study, AMPK activation was shown to attenuate cancer cell death under energy stress conditions through FAO-induced NADPH production [65].

In human breast cancer MCF-7 cells, hypoxia induces HIF-1α-dependent accumulation of lipid droplets and FAO, thereby providing reducing power required for protecting these cells from oxidative stress [66]. Inhibition of FAO by etomoxir, a carnitine palmitoyltransferase 1 inhibitor, markedly reduced cellular ATP levels and viability of human glioblastoma cells [67,68] and human leukemia cells [67,68]. In addition, inhibition of FAO increased the intracellular ROS accumulation while decreasing the levels of NADPH and GSH. These findings suggest that inhibition of FAO may cause cell death and ATP depletion [67]. Treatment with trimetazidine, another FAO inhibitor, resulted in a concentration-dependent decrease in cellular ATP levels and the induction of apoptosis in a murine lung alveolar carcinoma cell line, corroborating the above supposition [69].

Reductive carboxylation of glutamine mediated by IDH1 facilitated the transfer of reducing power, in the form of NADPH, from the cytosol to the mitochondria to help detoxify ROS generated in tumor cells grown in spheroids [70]. Gain-of-function mutations in the IDH1 gene occurs in various human cancers, including colorectal cancer [71] and glioma [72], which disrupt NADPH homeostasis by consuming NADPH for 2-hydroxyglutarate (2-HG) synthesis. 2-HG production competes with reductive biosynthesis and buffering of oxidative stress, both requiring NADPH [73]. Continuous production of 2-HG sensitizes colon cancer cells to oxidative stress. The consumption of NADPH by mutant R132H IDH1 may account for a metabolic vulnerability that sensitizes tumor cells to ionizing radiation [73]. IDH ablation diminished NADPH levels in transformed glioma cells and, when combined with a receptor tyrosine kinase inhibitor cocktail, caused a reduction in GSH with a concomitant increase in ROS and apoptosis [74]. These findings suggest that IDH1 upregulation represents a common metabolic adaptation of cancer cells to support macromolecular synthesis, aggressive growth, and therapeutic resistance.

### 5.2. Glutaminolysis

**Figure 5:**
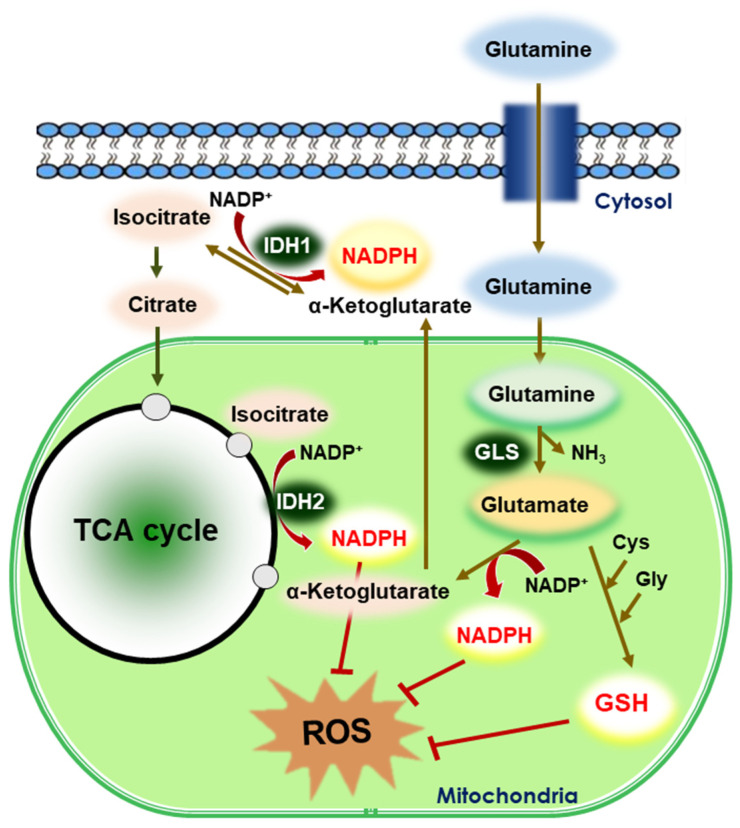
Reductive stress in glutaminolysis. Glutaminolysis is a metabolic pathway that involves the deamination of glutamine by GLS, yielding glutamate and ammonia. Glutamate is then converted to α-ketoglutarate, a TCA cycle intermediate. Glutamine supports the production of GSH and NADPH, which are two major reducing powers in the cells. IDHs catalyze the oxidative decarboxylation of isocitrate to α-ketoglutarate and reduce NADP^+^ to NADPH. In general, IDH1 is predominantly present in the cytosol and IDH2 is localized in the mitochondria. The generated GSH and NADPH mitigate intracellular ROS production.

Glutamine is a nonessential amino acid that plays a key role in tumor metabolism, serving as a source of carbon and nitrogen for biosynthetic processes, an intermediate for energy production, and a precursor for GSH synthesis [75]. Glutamine metabolism is used by cancer cells not only to circumvent the hypoxic/oxidative stress in the tumor microenvironment but also to resist targeted therapy (Figure 5). Increased glutamine catabolism is a common feature of tumor metabolic reprogramming by which cancer cells support their proliferation, signal transduction, and redox homeostasis [76]. Therefore, targeting glutamine metabolism holds a great potential for use as an anticancer strategy, and has been the focus of recent anticancer research [77]. Glutamine deprivation lowers the levels of GSH in neuroblastoma cells, resulting in altered redox balance, decreased cell proliferation, and increased chemosensitivity to the alkylating chemotherapeutic agent, melphalan [78]. Goto et al., reported that inhibition of glutaminolysis was associated with the depletion of intracellular GSH content and subsequent ROS generation, particularly in HL-60 promyelocytic leukemia cells that are dependent on glutamine metabolism [79].

Reductive glutamine metabolism has been reported to be highly dependent on the function of cytosolic IDH1, because the amount of [3-^2^H] glucose, which labels cytosolic NADPH, was reduced in lung cancer (H460) cells nullified for IDH1 [80]. In this study, overexpression of IDH1 was shown to mitigate mitochondrial ROS production in spheroids. In contrast, suppressing IDH1 reduced spheroid growth through a mechanism involving mitochondrial ROS [80]. Isocitrate/citrate produced in the cytosol enters the mitochondria and participates in oxidative metabolism. NADPH generated in the mitochondria helps cells counteract excessive mitochondrial ROS accumulation and maximize cell growth. Adaptation to anchorage independence requires a change in citrate metabolism, initiated by IDH1-dependent reductive carboxylation and culminating in the suppression of mitochondrial ROS [80].

Mutations in oncogenes promote increased glutamine utilization for the production of reducing equivalents [77,81]. The oncogenic transcription factor c-Myc promotes glutaminolysis to stimulate the growth and proliferation of cancer cells through upregulation of glutaminase (GLS) [82]. In addition, the glutamine metabolic pathway is activated by K-Ras oncoprotein in pancreatic cancer [83]. Three different enzyme targets within the glutaminolysis pathway can be blocked to specifically inhibit proliferation of malignant cells [83]. Activation of the mTORC1 pathway as a result of cancer-associated mutations stimulates glutamine anaplerosis and cell proliferation by repressing Sirt4 transcription [84]. Conversely, treatment of glioblastoma multiforme cells with mTOR inhibitors enhanced GLS1 expression and utilization of glutamine [85]. Knockdown of GLS1 significantly reversed the resistance to mTOR-targeted treatment in these cells [85]. Elevated transport of glutamine through L-glutamine carrier proteins, such as SLC1A5 and SLC38A2, can promote resistance of breast cancer cells to paclitaxel [86].

Cancer cells grown in suspension utilize reductive carboxylation of glutamine to transfer reducing power from the cytosol to the mitochondria, which detoxifies ROS and promotes anchorage-independent growth and survival [80]. Reductive glutamine metabolism provides citrate and acetyl-CoA for lipid synthesis during hypoxia [87,88,89]. Reductive carboxylation of glutamine to generate citrate requires the consumption of NADPH, which is predicted to exacerbate ROS accumulation.

## 6. Role of Redox-Regulated Signaling Molecules in Reductive Stress-Associated Tumorigenesis

**Figure 6:**
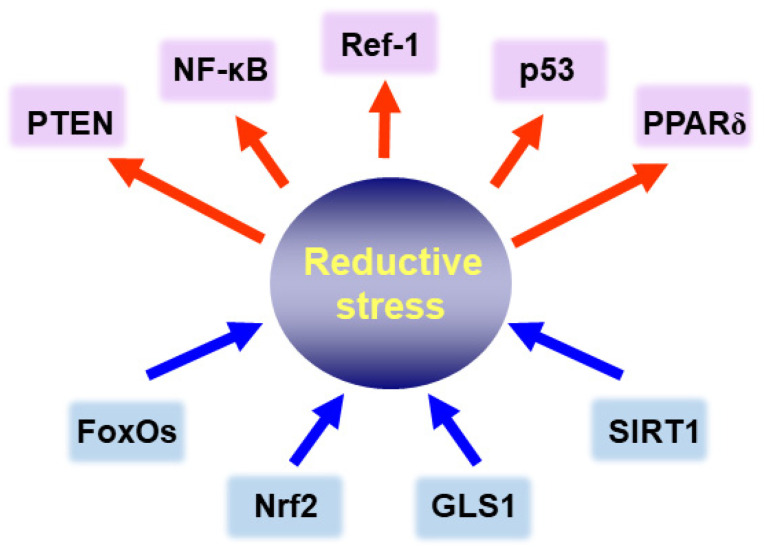
Intracellular signaling molecules modulating reductive stress in cancer cells. Blue arrows are signaling proteins that can induce reductive stress, and red arrows represent redox-sensitive proteins involved in cell growth and survival.

It is well known that the equilibrium between molecular redox duos, such as GSH/GSSG, NADPH/NADP or cysteine/oxidized form of cysteine (disulfide), is essential for regulating various signaling processes responsible for maintaining intracellular homeostasis [90,91,92]. The overall redox status of a cell, in particular, the cytoplasmic versus nuclear thiol balance affects the activity of signaling molecules. Some transcription factors and related molecules associated with the reductive stress in cancer cells are depicted in Figure 6, and their functions are described in the following sections.

### 6.1. Peroxisome Proliferator-Activated Receptor δ (PPAR δ)

PPARδ plays an important role in energy homeostasis by modulating glucose and lipid metabolism [93,94]. In addition to its metabolic role, PPARδ is overexpressed in some human tumors. PPARδ overexpression has been reported to accelerate colorectal tumor growth and progression by upregulating expression of vascular endothelial growth factor [95]. It is speculated that increased PPARδ activity may contribute to reprogramming of glutamine metabolism and drug resistance in cancer cells [96].

Increased glutamine metabolism supports the survival of sorafenib-resistant hepatocellular carcinoma (HCC) cells via the NADPH-dependent GSH redox system [96]. Enhanced glutamine metabolism in sorafenib-resistant HCC cells accounts for enhanced activation of biosynthetic pathways, greater proliferative capacity, and increased resistance to oxidative stress. The increase in glutamine metabolism and reductive glutamine carboxylation in sorafenib-resistant HCC cells has been attributed to the upregulation of PPARδ. Inhibition of glutamine metabolism or PPARδ function reversed the metabolic reprogramming in sorafenib-resistant HCC cells and sensitized them to sorafenib [96]. Sorafenib-resistant HCC cells exhibited markedly elevated glutamine utilization and reductive glutamine metabolism, which drives cell proliferation and maintains redox balance [96]. In addition, PPARδ upregulation in breast cancer cells is associated with more aggressive clinical behavior [97]. Glucose deprivation is known to cause oxidative stress in cancer cells [98], and PPARδ has been shown to fortify antioxidant defense [99]. Upregulation of PPARδ protected human breast cancer cells cultured in low-glucose conditions from oxidative stress, which was attributed to augmented antioxidant defense signaling mediated in part by catalase [97].

### 6.2. Phosphatidylinositol 3-Kinase (PI3K)/Protein Kinase B (Akt)

The PI3K-Akt axis is vital in cell survival as well as proliferation, and its hyperactivation contributes to chemotherapy resistance in tumor cells [100]. Nrf2 as a central mediator of ROS detoxification is regulated by PI3K/Akt signaling. Activation of Nrf2 signaling through Akt-mediated phosphorylation on a specific serine residue stimulates cancer cell survival by providing protection against excessive oxidative stress [101]. In BRCA1-deficient cells, estrogen promotes cancer cell survival through PI3K/Akt-mediated Nrf2 activation, which protects them from ROS-induced death and allows accumulation of mutations [102]. Conversely, when Akt signaling is blocked, ROS levels are reduced due to both decreased mitochondrial activity and increased FoxO-mediated expression of sestrin 3. This mechanism is responsible for resistance to ROS-mediated senescence and apoptosis in cancer cells [103]. Therefore, the association between Akt signaling and ROS metabolism is likely to be context-dependent and influenced by the down-stream target molecules involved.

### 6.3. Phosphatase and Tensin Homolog (PTEN)

The tumor suppressor PTEN is a negative regulator of the PI3K-Akt signaling. The expression levels, protein conformation, and subcellular localization of PTEN are regulated by a variety of genetic, epigenetic, post-transcriptional, and post-translational mechanisms [104]. Functional inactivation of PTEN, has been frequently observed in diverse human malignancies [105]. The catalytic activity of PTEN can be controlled by intracellular redox status. For instance, PTEN undergoes reversible oxidation by hydrogen peroxide, which affects its tyrosine phosphatase activity [106]. The cysteine 124 and the nearby cysteine 71, in the 3-dimensional structure of PTEN, form a disulfide bond in an oxidizing environment [107]. In particular, the Cys124 residue present in the catalytically active site of PTEN is highly sensitive to oxidation and forms a disulfide bond with the Cys71 residue in the presence of ROS, which permits reversible inactivation of the enzyme [108]. GSH [109] and several redox-effector proteins, including peroxiredoxin 1 [110], thioredoxin 1 [111], and glutaredoxin 5 [112] have been reported to promote PTEN catalysis in cells by keeping it in a reduced form, and to reactivate the oxidized enzyme. In some circumstances, however, oxidative modifications are irreversible, and PTEN may not be able to restore its catalytic activity even under reducing conditions [113]. The catalytic P-loop region containing the cysteine 124 residue is highly susceptible to modification, and hence constitutes a hot spot for tumor-related mutations [114].

### 6.4. Redox Factor-1 (Ref-1)

Ref-1 plays a vital role in the base excision repair. It also participates in intracellular signal transduction by virtue of maintaining redox-sensitive transcription factors (e.g., NF-κB, p53, STAT3, AP-1, and HIF-1α) in a functionally active reduced state [115]. While the C-terminal domain of Ref-1 retains the endonuclease activity responsible for DNA repair, the N-terminal domain is indispensable for its redox activity [116,117,118]. Ref-1 stimulates the DNA binding activity of some of aforementioned transcription factors by preventing oxidation of specific cysteine residues and/or reactivating the oxidized form. For instance, Ref-1 enhances DNA binding activity of the p53 tumor suppressor protein by keeping cysteine residues located within the central DNA-binding domain in a reduced state [116]. Likewise, STAT3 binds to DNA more effectively in the presence of Ref-1, and concurrent blockade of STAT3 and Ref-1 significantly inhibits the migration of pancreatic cancer cells [119]. In contrast, the redox function of Ref-1 negatively regulates Nrf2 [120]. Therefore, combined inhibition of HO-1 and Ref-1 redox activity has been shown to synergistically repress the proliferation of pancreatic cancer cells. APX3330, a specific Ref-1 redox inhibitor, retards tumor growth and progression with limited toxicity in both in vitro and in vivo models [120,121].

### 6.5. Forhead Box O (FoxO)

FoxO proteins belong to a subfamily of forkhead transcription factors that participate in cell fate determination. So far, there are four isoforms that have been identified in mammals: FoxO1, FoxO3a, FoxO4, and FoxO6. Functions of FoxO proteins are regulated by ROS and implicated in the regulation of intracellular redox status [122]. Under conditions of oxidative stress, acetylated FoxO3a migrates from the cytosol into the nucleus, where it may interact with nuclear sirtuin 1 (SIRT1) to become deacetylated [123]. SIRT1, a sensor of the cellular redox status, is activated when the supply of reducing equivalents, especially NADH, is limiting [124]. Treatment of SIRT1 protein with physiologically relevant concentrations of exogenous GSH restored the SIRT1 deacetylase activity, which was attenuated by cysteine-*S*-nitrosation [125]. Deacetylated FoxOs become enriched in the nucleus and upregulates the transcription of genes involved in cell cycle arrest and DNA repair as well as antioxidant enzymes such as MnSOD [126,127]. In addition, FoxOs are indispensable for SIRT1-dependent cancer cell survival. Nuclear localization of FoxO1 was observed in tamoxifen-resistant MCF-7 cells, which canonically overexpress multidrug resistance protein 2 (MRP2) protein [128]. The proximal promoter region of the human MRP2 gene contains four putative FoxO binding sites, and MRP2 gene transcription was stimulated by FoxO1 overexpression in MCF-7 cells. Nuclear localization of FoxO1 is regulated by SIRT1 deacetylase activity, which accounts for the upregulation of MRP2 in tamoxifen-resistant MCF-7 cells [128].

## 7. Concluding Remarks

In general, intracellular ROS levels are higher in cancer cells than in normal counter parts. Excessive ROS is detrimental to cancer cells as well as normal cells. Cancer cells protect themselves from oxidative stress through upregulation of multiple antioxidant genes, which buffers ROS levels for survival and growth. Cancer cells also exhibit metabolic alterations to manage oxidative stress. In this regard, the GSH synthetic pathway is a promising therapeutic target [129]. Nrf2 is a key regulator of production, recycling, and utilization of GSH [130]. In addition, a growing body of data suggests that constitutive or sustained hyperactivation of Nrf2 and subsequent overexpression of antioxidant proteins, including HO-1, contribute to the manifestation of a malignant phenotype in various tumors. Antioxidant-induced reductive stress constitutes an essential part of the evolutionarily conserved cellular defense that counteracts a wide array of endogenous and exogenous stressors, many of which generate excessive ROS. Reductive stress is exploited by cancerous or transformed cells for their growth advantage and to acquire tolerance/resistance to chemotherapy and radiotherapy. Furthermore, understanding the reductive stress underlying the regulation of antioxidant enzymes and metabolic pathways in the tumor microenvironment will provide novel therapeutic approaches in the management of cancer and resistance to anticancer drugs. In this context, it is important to understand the transition as well as the formation of metabolites under oxidative and reductive stresses in the tumor microenvironment. Recently, Metere et al. have reported that oxidative stress provokes the alteration of the metabolic profile in thyroid cancer cells [131]. Through metabolomic analysis, this study revealed increases in lactate and aromatic amino acids, such as tyrosine and phenylalanine, and an average decrease in citric acid in thyroid cancer tissue compared to healthy tissue [131]. Furthermore, accumulation of acetate and formic acid may be attributable to a decrease in activity of the citric acid cycle, and a shift towards glycolysis in cancer tissue [131]. Further studies are warranted to explore the metabolic reprograming of other cancer cells under oxidative versus reductive stress in relation to their growth and survival.
